# High prevalence of thyroid nodules and their association with cardiometabolic risk in Mongolia

**DOI:** 10.3389/fcvm.2026.1762003

**Published:** 2026-06-08

**Authors:** Oyuntugs Byambasukh, Anar Bayarmunkh, Tsakhim-Erdene Tsendjav, Tugsjargal Purevsukh, Tuvshinjargal Dashjamts, Batzorig Bayartsogt, Enkhtur Yadamsuren, Altaisaikhan Khasag, Oyunsuren Enebish, Tumur-Ochir Tsedev-Ochir

**Affiliations:** 1 Department of Endocrinology, School of Medicine, Mongolian National University of Medical Sciences, Ulaanbaatar, Mongolia; 2 Department of Radiology, School of Medicine, Mongolian National University of Medical Sciences, Ulaanbaatar, Mongolia; 3 Department of Epidemiology and Biostatistics, School of Public Health, Mongolian National University of Medical Sciences, Ulaanbaatar, Mongolia; 4 Mongolian National University of Medical Sciences, Ulaanbaatar, Mongolia; 5 Ministry of Health of Mongolia, Ulaanbaatar, Mongolia; 6 State Central Third Hospital, Ulaanbaatar, Mongolia

**Keywords:** cardiovascular diseases, diabetes, health checkups, Mongolia, obesity, thyroid nodules

## Abstract

**Introduction:**

Mongolia faces rising rates of obesity, diabetes, and cardiovascular disease, yet little is known about their relationship with thyroid nodules. This study aimed to assess the prevalence of thyroid nodules and their association with cardiometabolic outcomes in the Mongolian population.

**Methods:**

A nationwide retrospective analysis was conducted among 36,395 adults who underwent thyroid ultrasound during routine health checkups. Demographic, anthropometric, and metabolic parameters were recorded, including body mass index (BMI), central obesity, blood pressure, diabetes, and cardiovascular risk based on WHO criteria.

**Results:**

Thyroid nodules were identified in 40.3% (*n* = 14,651) of participants. Age-adjusted prevalence was 32.1% overall, 19.8% in men, and 39.2% in women, with higher rates in Ulaanbaatar (43.9%) than in rural areas (35.4%). Participants with nodules had higher BMI, central obesity, diabetes, and cardiovascular risk compared to those without nodules. Prevalence was greatest in the very high CVD risk category (60.0% in women, 33.1% in men). Multivariate models confirmed independent associations between thyroid nodules and age, female sex, obesity, and cardiovascular risk.

**Conclusions:**

Thyroid nodules are highly prevalent in Mongolia and cluster with adverse cardiometabolic outcomes. Nodules may serve as a clinical marker of elevated cardiovascular risk. Future longitudinal studies should explore potential bidirectional pathways between obesity, thyroid nodules, and cardiometabolic disease.

## 1 Introduction

Thyroid nodules are increasingly diagnosed in clinical practice, primarily due to advancements in imaging techniques that enable the detection of small and asymptomatic nodules (1–3). Studies have highlighted a correlation between improved healthcare access and a rising incidence of thyroid nodules and thyroid cancers (4). Earlier single-center studies conducted in the mid-2000s reported prevalence rates of thyroid nodules ranging from 28.3% to 42.4% in asymptomatic women and 14.1% to 29.1% in men (5–8). Similar findings have been reported in countries such as China and Korea. For example, a large-scale health checkup study in Korea reported a prevalence of thyroid nodules and cysts of 34.2% among 72,319 participants who underwent thyroid ultrasound between 2004 and 2010 (9). In China, a cross-sectional study involving over 120,000 participants reported a prevalence of 34.1% for thyroid nodules in the Chongqing population (10). Additionally, European studies have shown that thyroid nodules are present in approximately 33% of the general population, as detected by high-frequency ultrasound, indicating a high prevalence even in seemingly healthy individuals (1).

The increased detection rate is widely considered a key factor contributing to the rising prevalence of thyroid nodules. The prevalence is influenced by various factors, including detection methods, sex, age, iodine intake, and radiation exposure (3–6, 11–13). Recently, metabolic parameters have gained attention as significant risk factors. Research has shown associations between insulin resistance and an increased prevalence of thyroid nodules in both iodine-deficient and iodine-sufficient regions (4, 13, 14). Additionally, hypertension and diabetes have been reported as independent risk factors for thyroid nodules (14–19).

In recent years, Mongolia has seen a sharp rise in obesity, diabetes, and metabolic syndrome, all of which are known risk factors for thyroid nodules. Combined with possible iodine deficiency, these trends suggest a substantial burden of thyroid nodules in the population (20–23). The rising prevalence of these metabolic disorders in Mongolia therefore points to a potentially higher burden of thyroid nodules, which may be compounded by suspected iodine deficiency in the region. Although studies have documented thyroid nodule prevalence in other Asian countries, comprehensive data from Mongolia—particularly in relation to cardiometabolic factors—remain limited.

To address this knowledge gap, we conducted a large-scale, multicenter study involving 36,395 participants who underwent thyroid ultrasound during routine health checkups across Mongolia. The primary aim was to determine the prevalence of thyroid nodules and explore their association with cardiometabolic risk factors in the Mongolian population.

## 2 Material and methods

### 2.1 Data collection and study participants

This study utilized data from a national health screening program conducted by the Ministry of Health, Mongolia, from 2022 to 2023, covering all regions across the country. The participants included individuals screened during this period, with the screening being conducted in diverse family centers, ensuring a representative sample from both rural regions and the capital city. The methodology for the screening and data collection has been previously described in detail (24).

A total of 48,576 adults underwent thyroid ultrasound examination during the study period. Participants with incomplete data on key cardiometabolic variables or missing ultrasound results were excluded (*n* = 11,888). Individuals with a prior history of thyroid cancer or previous thyroid surgery were also excluded to ensure a homogeneous screening population and to minimize bias related to altered thyroid anatomy or pre-existing disease. The final analytical cohort consisted of 36,395 participants. The proportion of missing data was low and considered unlikely to materially influence the results.

This study was conducted following the principles outlined in the Declaration of Helsinki and received ethical approval from the Ministry of Health, Mongolia (Approval No: 23/042, dated July 5, 2023).

### 2.2 Thyroid ultrasound assessment

High-resolution ultrasound machines with 7.5 to 12 MHz or 8 to 15 MHz linear transducers were used for the thyroid evaluations. A team of five researchers carefully reviewed and extracted thyroid characteristics from ultrasound reports. To ensure data accuracy, cross-validation was performed by randomly rechecking records among the researchers. Any inconsistencies were resolved through collaborative review and re-testing.

Thyroid nodules were defined as discrete lesions within the thyroid gland, distinguishable from surrounding parenchyma. Both solid nodules and cystic lesions were included in the analysis and categorized together as “thyroid nodules”, reflecting real-world screening practice. Detailed sonographic characteristics, including nodule size, number, and malignancy risk features, were not consistently available and were therefore not included in the analysis.

### 2.3 Demographic and cardiometabolic parameters

Demographic data included age, gender, living area (categorized into four regions and the capital city), education level, and smoking status (including current daily users and recent quitters within six months). Cardiometabolic parameters included BMI, waist circumference, blood pressure, lipid profile (total cholesterol), glucose levels, CVD risk, and diabetes status. BMI was classified as normal weight, overweight (25–29.9 kg/m²), or obese (≥30 kg/m²). Central obesity was defined as a waist circumference of ≥90 cm for men and ≥80 cm for women. Diabetes was defined based on fasting plasma glucose levels (≥126 mg/dL) or the use of antidiabetic medications.

CVD risk was assessed using the WHO risk prediction charts for the Asia region. The risk assessment took into account age, gender, systolic blood pressure, smoking status, and diabetes status, categorizing participants into low (<10%), moderate (10%–19%), high (20%–29%), or very high (≥30%) CVD risk groups. The WHO cardiovascular risk model used does not include lipid parameters. Participants who reported a history of stroke or myocardial infarction were automatically placed in the very high-risk category.

### 2.4 Statistical analysis

Continuous variables were reported as means with standard deviations, while categorical variables were presented as numbers with percentages. Group comparisons were made using Student's *T*-test for continuous variables and Pearson's chi-square test for categorical variables. Continuous variables were tested for normality using the Kolmogorov–Smirnov test. Normally distributed variables are reported as mean ± SD, while skewed variables are additionally presented as median (IQR).

Age-adjusted thyroid nodule prevalence rates were calculated using direct standardization. Age categories were defined as “Below 35”, “35–55”, and “Above 55” years, with prevalence rates weighted according to a hypothetical standard population distribution (30% for “Below 35”, 40% for “35–55”, and 30% for “Above 55”). Additionally, age-adjusted prevalence rates were calculated for different regions (Western, Khangai, Central, Eastern, and Ulaanbaatar), separately for men, women, and the total population. Regional differences were displayed visually using distinct labeling.

Logistic regression models were used to assess associations between demographic and metabolic factors and thyroid nodules, with odds ratios (ORs) and 95% confidence intervals (CIs) calculated. Subgroup analyses examined thyroid nodule prevalence across categories of age, gender, BMI (normal weight, overweight, obese), and CVD risk (low, moderate, high/very high). BMI and central obesity were analyzed separately in regression models to avoid collinearity. For regression, BMI was used as a continuous variable, while categorical obesity status is shown only in descriptive tables. Blood pressure and diabetes risk were consistently presented across descriptive and regression analyses. Given the relatively high prevalence of thyroid nodules in the study population, odds ratios may overestimate the strength of associations and were interpreted with caution.

Variance inflation factors (VIFs) were calculated to assess multicollinearity, and no significant collinearity was observed between BMI and central obesity. Bonferroni correction was applied based on the number of pairwise comparisons performed, and adjusted significance thresholds were reported accordingly.

All analyses were performed using IBM SPSS V.22.0 and GraphPad Prism V.4.03. Statistical significance was set at *p* < 0.05.

## 3 Results

**Figure 1:**
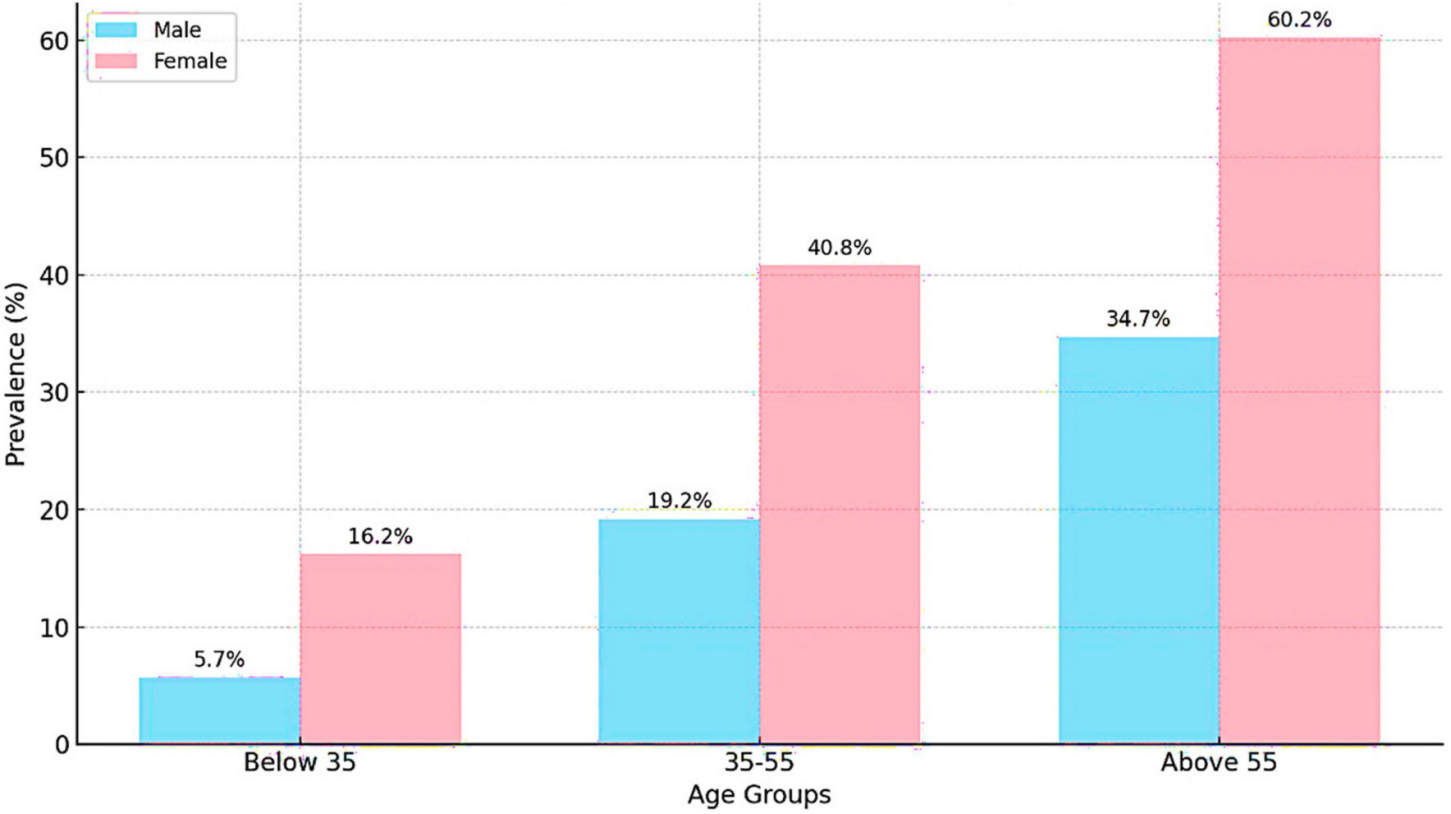
Prevalence of thyroid nodules by age and gender.

Among the participants, 40.3% (*n* = 14,651) were found to have thyroid nodules. The proportion was higher in women (76.7%, *n* = 11,242) compared to men (23.3%, *n* = 3,409) (*p* < 0.001). The age-adjusted prevalence rates were 32.1% overall, 19.8% in men, and 39.2% in women. The prevalence of thyroid nodules increased steadily with age, ranging from 15.5% in the youngest group to 53.7% in the oldest group in the total study population, with women consistently showing higher prevalence across all age groups, as illustrated in Figure 1.

**Figure 2:**
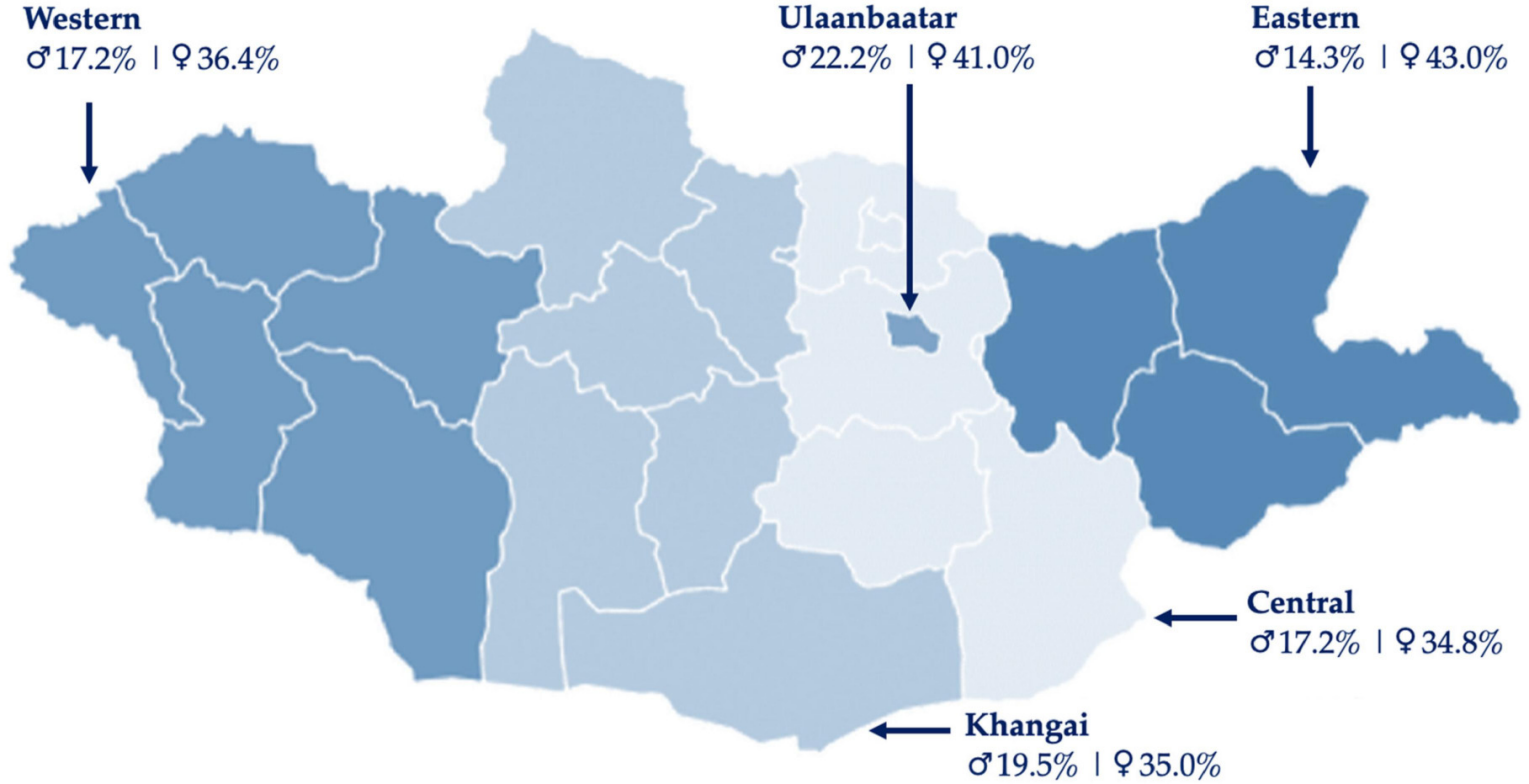
Age-adjusted prevalence of thyroid nodules by region.

Regarding regional distribution, subjects living in Ulaanbaatar had a higher prevalence (43.9%, *n* = 10,703) compared to those in rural areas (35.4%, *n* = 8,363) (*p* < 0.01). However, after adjusting for age and weighting prevalence rates across regions, these differences were not substantial, suggesting that the observed variation was largely explained by population structure (Figure 2).

Table 1 presents the general characteristics of the study population stratified by thyroid nodule status. Participants with nodules were older (mean age: 56.56 ± 10.1 years) compared to those without nodules (51.83 ± 10.1 years, *p* < 0.001). The nodular group also had a higher mean BMI (27.91 ± 4.7 kg/m² vs. 26.77 ± 4.7 kg/m², *p* = 0.002) and a greater prevalence of central obesity (61.1% vs. 45.9%, *p* = 0.002). Cardiovascular parameters showed modest but statistically significant differences between groups. Participants with nodules had slightly higher systolic blood pressure (125.24 ± 16.2 vs. 123.3 ± 16.3 mmHg, *p* = 0.018) and diastolic blood pressure (81.05 ± 10.6 vs. 80.05 ± 10.67 mmHg, *p* < 0.001). Total cholesterol levels were also higher in the nodular group (5.34 ± 1.64 vs. 5.21 ± 1.39 mmol/L, *p* = 0.003) (Table 1). Fasting glucose levels were marginally higher in participants with nodules (5.35 ± 1.63 vs. 5.30 ± 1.53 mmol/L, *p* < 0.001), although the absolute difference was small. The prevalence of diabetes was higher in the nodular group (4.3% vs. 2.6%, *p* < 0.001). In addition, overweight and obesity were more common among participants with nodules (68.4% vs. 54.0%, *p* < 0.001) (Table 1). With respect to cardiovascular risk, individuals with thyroid nodules were more frequently classified into higher risk categories. The proportion of participants in the high or very high-risk group was greater in the nodular group compared to the non-nodular group (14.0% vs. 10.4%, *p* < 0.001), while the proportion in the low-risk group was slightly lower (82.9% vs. 84.9%) (Table 1).

**Table 1:** Baseline characteristics of the study population according to thyroid nodule status.

Findings	Non-nodular (*n* = 21,744)	Nodular (*n* = 14,651)	*p*-value
Age (year)	51.83 ± 10.1	56.56 ± 10.1	<0.001
Sex			
Men, *n* (%)	9,798 (45.1)	3,409 (76.7)	<0.001
Women, *n* (%)	11,946 (54.9)	11,242 (23.3)	<0.001
Obesity status			
BMI (kg/m²)	26.77 ± 4.7	27.91 ± 4.7	0.002
Overweight-obesity, *n* (%)	11,742 (54)	10,021 (68.4)	<0.001
Central obesity, *n* (%)	9,970 (45.9)	8,952 (61.1)	0.002
Diabetes status			
Fasting glucose (mmol/L)	5.3 ± 1.53	5.35 ± 1.63	<0.001
Low risk, *n* (%)	18,267 (84.9)	12,050 (82.9)	<0.001
Moderate to high risk, *n* (%)	2,320 (10.7)	1,719 (11.8)	<0.001
Diabetes, *n* (%)	935 (2.6)	759 (4.3)	<0.001
Cardiovascular risk			
Systolic BP (mmHg)	123.3 ± 16.3	125.24 ± 16.2	0.018
Diastolic BP (mmHg)	80.05 ± 10.67	81.05 ± 10.6	<0.001
Cholesterol (mmol/L)	5.21 ± 1.39	5.34 ± 1.64	0.003
Low CVD risk, *n* (%)	10,700 (50.2)	6,087 (42.2)	<0.001
Moderate CVD risk, *n* (%)	8,393 (39.4)	6,310 (43.8)	<0.001
High/very high CVD risk, *n* (%)	2,232 (10.4)	2,024 (14)	<0.001

Data are presented as mean ± standard deviation (SD) or number (percentage), as appropriate. *P*-values were calculated using Student's *t*-test for continuous variables and chi-square test for categorical variables, with Bonferroni correction applied for multiple comparisons. BMI, body mass index; BP, blood pressure; CVD, cardiovascular disease; SD, standard deviation.

As shown in Table 2, both univariate and multivariate analyses demonstrated that age, sex, adiposity-related measures, and cardiovascular risk were associated with thyroid nodules. Age remained a strong predictor, with each additional year associated with a 4.9% increase in odds (multivariate OR: 1.049, 95% CI: 1.046–1.051, *p* < 0.001). Female sex was also strongly associated, with women having more than threefold higher odds compared to men (multivariate OR: 3.055, 95% CI: 2.903–3.215, *p* < 0.001). Body mass index and waist circumference were both associated with thyroid nodules, although the magnitude of association in the adjusted model was modest. Diabetes was not significantly associated after adjustment (multivariate OR: 1.041, 95% CI: 0.935–1.159, *p* = 0.468). Cardiovascular risk categories remained significantly associated with thyroid nodules. Compared with the low-risk group, individuals in the moderate-risk category had higher odds (multivariate OR: 1.101, 95% CI: 1.047–1.157, *p* < 0.001), as did those in the high or very high-risk group (multivariate OR: 1.120, 95% CI: 1.037–1.211, *p* = 0.004) (Table 2).

**Table 2:** Association between cardiometabolic factors and thyroid nodules.

Findings	Univariate analysis			Multivariate analysis		
	OR	95% CI	*P*-value	OR	95% CI	*P*-value
Age (year)	1.046	1.044–1.049	<0.001	1.049	1.046–1.051	<0.001
Sex						
Men	1.0			1.0		
Women	2.689	2.566–2.818	<0.001	3.055	2.903–3.215	<0.001
Obesity status						
BMI, kg/m^2^	1.027	1.022–1.031	<0.001	1.015	1.008–1.022	<0.001
Normal weight	1.0					
Overweight	1.220	1.159–1.284	<0.001	-		
Obesity	1.366	1.292–1.444	<0.001	-		
Waist circumference, cm	1.004	1.003–1.006	<0.001	1.005	1.003–1.008	<0.001
Non-central obesity	1.0					
Central obesity	1.516	1.449–1.587	<0.001	-		
Diabetes status						
Without DM	1.0			1.0		
With DM	1.210	1.097–1.335	<0.001	1.041	0.935–1.159	0.468
Cardiovascular risk						
Systolic BP (mmHg)	1.007	1.006–1.009	<0.001	–		
Low CVD risk	1.0			1.0		
Moderate CVD risk	1.323	1.264–1.385	<0.001	1.101	1.047–1.157	<0.001
High/very high CVD risk	1.598	1.493–1.711	<0.001	1.120	1.037–1.211	0.004

Data are presented as odds ratios (ORs) with 95% confidence intervals (CIs) from univariate and multivariate logistic regression analyses. Reference categories are indicated for categorical variables. BMI and waist circumference were evaluated in separate multivariate models due to potential collinearity and were not included simultaneously. BMI, body mass index; BP, blood pressure; CVD, cardiovascular disease; DM, diabetes mellitus.

**Figure 3:**
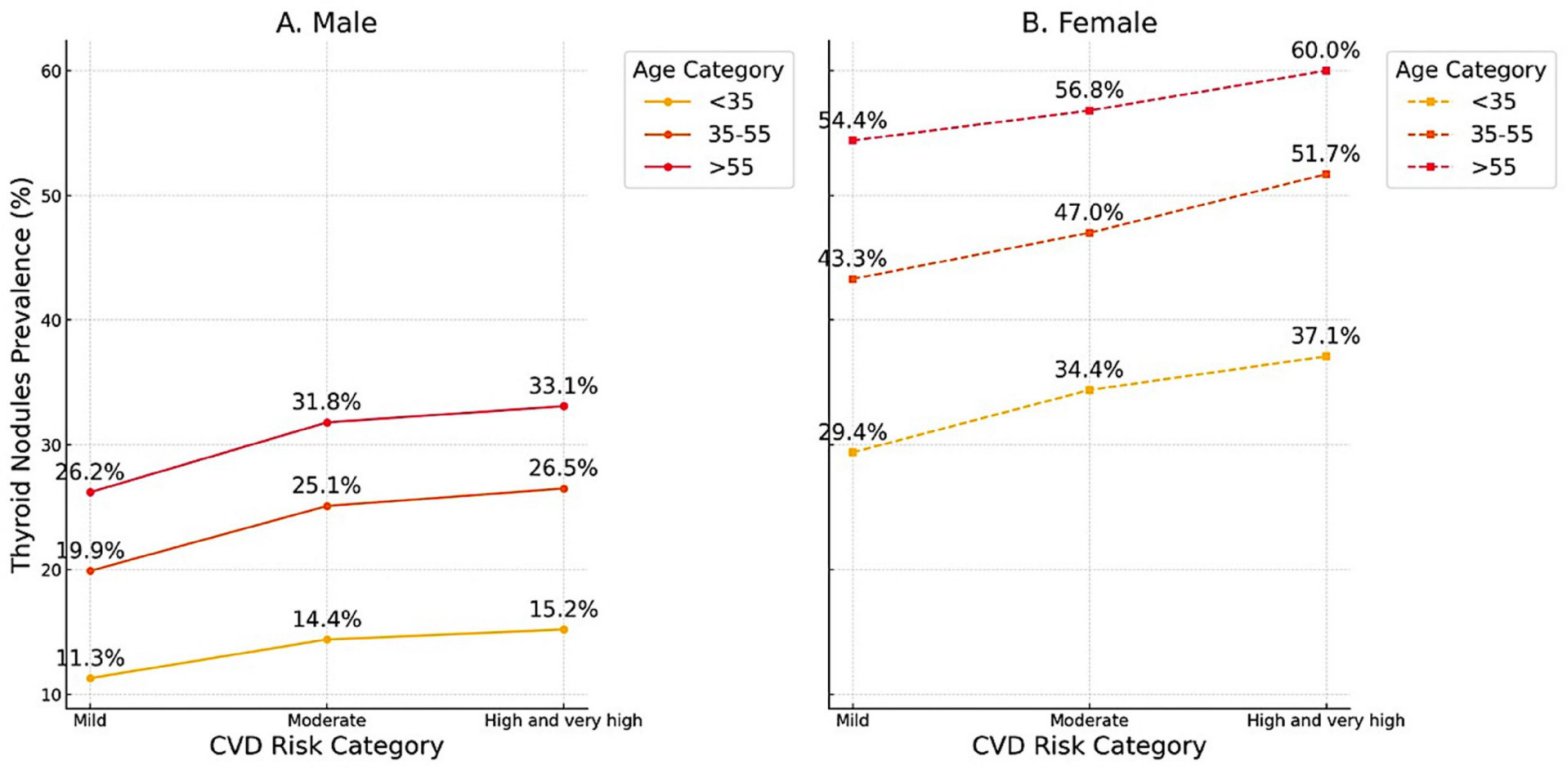
Prevalence of thyroid nodules by cardiovascular risk category stratified by age and gender.

Subgroup analysis showed that the prevalence of thyroid nodules increased with BMI across age groups in both men and women. For example, among men under 35 years, prevalence increased from 3.4% in the normal weight group to 9.6% in the obese group. In women aged over 55 years, prevalence ranged from 56.0% in the normal weight group to 62.9% in the obese group. Figure 3 shows the distribution of thyroid nodule prevalence across cardiovascular risk categories stratified by age and sex. In men, prevalence increased with higher cardiovascular risk, reaching 33.1% in the high/very high-risk group. A similar pattern was observed in women, where prevalence increased from 54.4% in the low-risk group to 60.0% in the high/very high-risk group.

## 4 Discussion

This nationwide study is the first large-scale investigation into the prevalence of thyroid nodules and their association with cardiometabolic risk factors in Mongolia. We found that the prevalence of thyroid nodules was 40.3%, with significant gender differences—76.7% in women and 23.3% in men. The age-adjusted prevalence rates were 32.1% overall, 19.8% in men, and 39.2% in women. These rates are higher than those reported in similar studies conducted in Korea (34.2%) and China (34.1%) (9, 10), suggesting a greater burden of thyroid nodules in the Mongolian population. The observed gender differences are consistent with trends noted in other regions, with women showing a much higher prevalence compared to men.

The higher prevalence among females may be related to hormonal influences, particularly estrogen, which has been shown to stimulate thyroid cell proliferation through estrogen receptor pathways (25, 26). Estrogen may also enhance the expression of growth factors that promote thyroid tissue expansion. In addition, genetic susceptibility and autoimmune mechanisms such as the higher prevalence of thyroid autoimmunity in women may contribute to this disparity (27, 28). More broadly, several mechanisms may underlie the association between thyroid nodules and cardiometabolic risk. Insulin resistance and chronic low-grade inflammation, commonly observed in obesity and metabolic syndrome, have been implicated in thyroid cell proliferation and nodule formation (14–16). Environmental exposures, including endocrine-disrupting chemicals, air pollution, and dietary factors, may also influence both thyroid function and cardiometabolic health (9, 10). These shared pathways may partly explain the co-occurrence of thyroid nodules with adverse metabolic profiles.

The age-related increase in thyroid nodule prevalence observed in this study is consistent with prior reports. This pattern may reflect cumulative exposure to environmental and metabolic risk factors over time, including radiation, chronic inflammation, and other environmental influences (29). As individuals age, the likelihood of prolonged exposure to such factors increases, which may contribute to progressive thyroid tissue changes.

Participants residing in urban areas exhibited a higher crude prevalence of thyroid nodules (43.9%) compared to those in rural areas (35.4%). However, after accounting for differences in age distribution, this variation was not substantial, suggesting that population structure largely explains the observed difference. Nevertheless, urban-related factors such as dietary patterns, environmental pollution, and lifestyle differences may still play a role and warrant further investigation. For example, exposure to endocrine-disrupting chemicals and dietary components that interfere with iodine metabolism could potentially influence thyroid health (30–32).

A key finding of this study is the association between thyroid nodules and cardiometabolic risk factors. Individuals with higher BMI, central obesity, and elevated cardiovascular risk were more likely to have thyroid nodules. Although diabetes was associated with thyroid nodules in univariate analysis, this association was not significant after adjustment, suggesting that the relationship may be mediated through other metabolic factors such as adiposity and overall cardiovascular risk. Cardiovascular risk categories remained associated with thyroid nodules in the multivariable model. However, it should be noted that the inclusion of a composite cardiovascular risk score alongside its component variables (such as age, blood pressure, and diabetes) may introduce conceptual overlap. Therefore, these findings should be interpreted with caution, as the cardiovascular risk variable likely reflects the combined effect of its underlying components rather than an entirely independent association. The observed patterns are consistent with previous studies reporting links between thyroid nodules and cardiometabolic factors (13–19). For example, community-based research in Beijing identified age, sex, and BMI as key factors associated with thyroid nodules (33). Mechanistically, hyperinsulinemia, insulin resistance, and chronic inflammation have been proposed as drivers of thyroid cell proliferation (34). Obesity, in particular, has been associated with increased leptin levels and low-grade inflammation, both of which may contribute to thyroid tissue growth (35). While these mechanisms are biologically plausible, the cross-sectional nature of our study does not allow determination of causality.

Our study has several strengths, including a large sample size and nationwide coverage, which enhance the robustness and generalizability of the findings within Mongolia. However, several limitations should be acknowledged. First, the cross-sectional design precludes conclusions regarding causality or temporal relationships. Second, iodine intake was not assessed, which is an important limitation given its known role in thyroid disease. Although iodized salt programs have been widely implemented in Mongolia, individual iodine status may still vary (36). Third, the dataset did not include detailed ultrasound characteristics such as nodule size, number, or risk features, limiting clinical interpretation. The inclusion of both solid and cystic lesions may also introduce heterogeneity in the outcome definition. Additionally, as the WHO cardiovascular risk model does not incorporate lipid profiles, cardiovascular risk may have been underestimated in some individuals. Given the inclusion of both individual cardiometabolic variables and a composite cardiovascular risk score, the observed associations should be interpreted as reflecting overlapping risk domains rather than fully independent effects. Finally, the study population was derived from voluntary health check-up programs, which may introduce selection bias, as participants could be more health-conscious than the general population.

These findings have potential public health implications. The high prevalence of thyroid nodules, particularly among women and older adults, highlights the importance of targeted screening strategies. Addressing modifiable risk factors such as obesity and cardiometabolic health may also be relevant in reducing the overall burden. However, given the observational nature of the data, these implications should be interpreted cautiously. Future research should focus on longitudinal designs to clarify causal pathways and explore the underlying biological mechanisms. Further studies incorporating iodine status, environmental exposures, and genetic factors would provide a more comprehensive understanding of thyroid nodule epidemiology in Mongolia.

## 5 Conclusion

This study provides the first nationwide evidence from Mongolia, demonstrating a high prevalence of thyroid nodules (40.3%) with marked gender and age differences. Importantly, the presence of thyroid nodules was strongly associated with adverse cardiometabolic outcomes, including obesity, diabetes, and high cardiovascular risk. These findings suggest that thyroid nodules may serve as a potential clinical indicator of increased cardiometabolic risk, rather than a causal factor.

Targeted strategies focusing on weight control, cardiovascular risk reduction, and early screening in high-risk groups are essential to reduce the dual burden of thyroid and cardiometabolic disease. Future longitudinal and mechanistic studies should clarify whether thyroid nodules actively contribute to cardiometabolic disease development or primarily act as an early marker of systemic metabolic dysfunction. Given the complex interplay observed in this study, it is also important to test for possible bidirectional pathways whereby obesity increases the likelihood of developing nodules, and once nodules are present, they may cluster with or exacerbate cardiovascular risk.

## Data Availability

The original contributions presented in the study are included in the article/Supplementary Material, further inquiries can be directed to the corresponding author/s.
